# PIO, A Large-Scale Dataset for Broiler Chicken Detection under Real Poultry Farming Conditions

**DOI:** 10.1038/s41597-026-07114-5

**Published:** 2026-04-09

**Authors:** Keyla Boniche, Edmanuel Cruz, José Carlos Rangel, Miguel Hidalgo-Rodríguez, Francisco Gómez-Donoso

**Affiliations:** 1 Facultad de Ingeniería Mecánica, Universidad Tecnológica de Panamá, Panamá City, 0819-07289 Panamá; 2 Centro Regional de Veraguas, Universidad Tecnológica de Panamá, Atalaya, 0901 Panamá; 3 Sistema Nacional de Investigación (SNI), SENACYT, Panamá City, 0816-02852 Panamá; 4 Facultad de Ingeniería de Sistemas Computacionales, Universidad Tecnológica de Panamá, Panamá City, 0819-07289 Panamá; 5 Institute for Computer Research, University of Alicante, 03080 Alicante, Spain

**Keywords:** Agriculture, Technology

## Abstract

Broiler chicken production is a cornerstone of global food security, yet monitoring animals in commercial farms remains challenging due to high stocking densities and variable environmental conditions. Progress in automated monitoring is hindered by the scarcity of domain-specific, publicly available datasets. To address this gap, we present Poultry Images for Object detection (PIO), a dataset designed to support the development and evaluation of computer vision models for poultry farming. PIO comprises 1,487 manually annotated images containing 327,289 instances of broiler chickens, collected from both commercial and prototype poultry houses across different growth stages. The dataset reflects realistic conditions such as variations in morphology, lighting and bird density. Annotations were generated using the LabelImg tool, with bounding boxes normalized to image dimensions for compatibility with state-of-the-art detection frameworks. To illustrate its utility, three YOLOv10 variants were trained and evaluated on PIO, demonstrating its suitability for benchmarking object detection models in precision livestock farming contexts, as shown in figure 1.

## Background & Summary

**Fig. 1:**
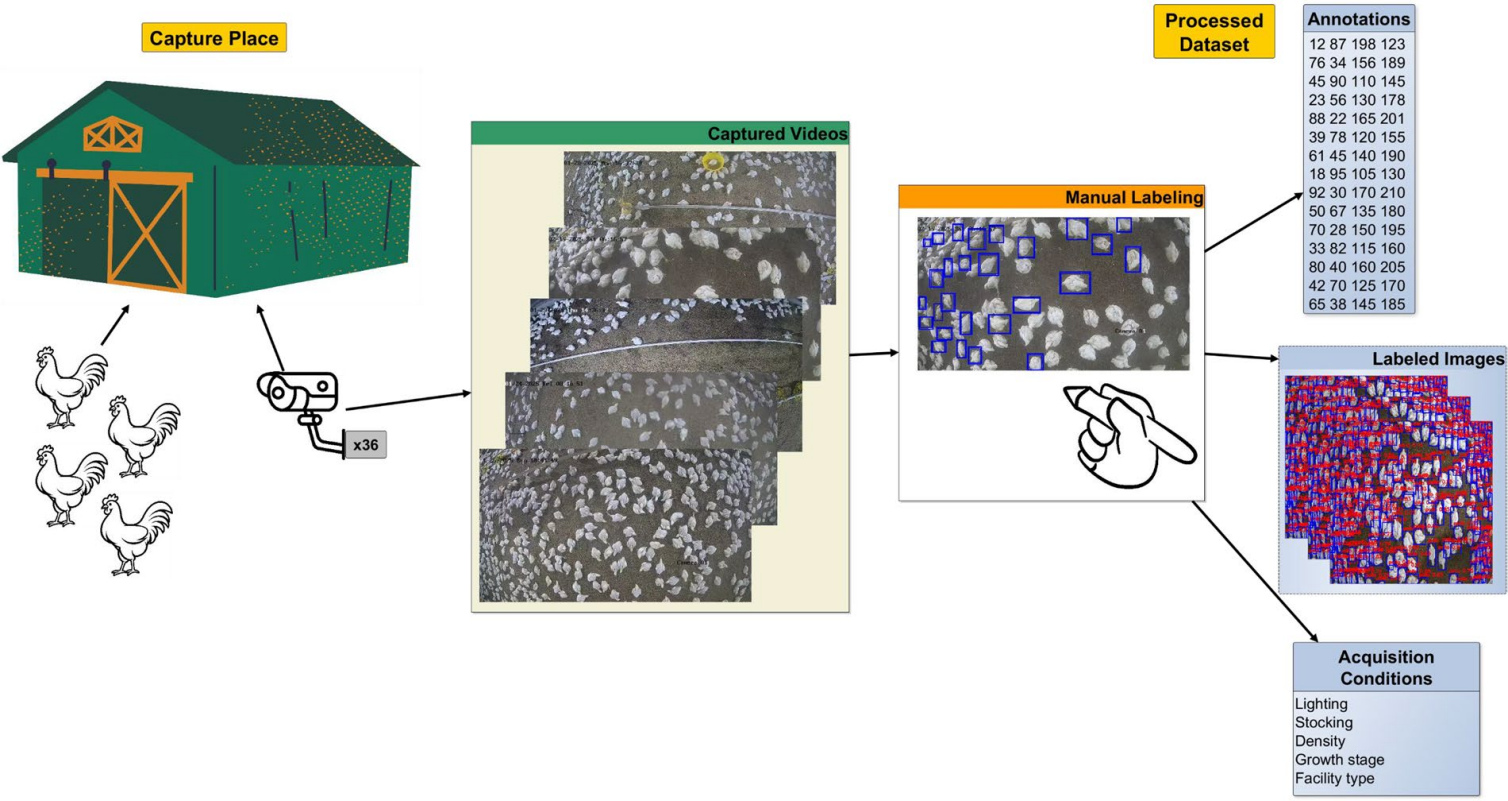
PIO Dataset Creation: Capture, manual annotation, and processing of 327,289 broiler chicken instances for precision poultry farming.

The growing global demand for poultry products underscores the urgent need to safeguard food security. Yet, despite significant technological progress in agriculture, modern broiler production continues to rely heavily on manual monitoring practices. These labor-intensive processes not only increase operational costs but also reduce accuracy and limit efficiency. As consumption continues to rise, the poultry industry faces a pivotal challenge: how to transition from manual oversight to automated, data-driven management. In this context, researchers have long recognized that many operations in poultry meat processing are physically and cognitively demanding. Automation in such environments provides the dual benefit of increasing productivity while improving the welfare of both workers and animals as shown in Fig. 1^1^.

At the same time, advances in sensing and computing have opened new possibilities for more precise and humane monitoring. Recent reviews of non-contact detection methods^2^—often powered by computer vision and deep learning—have shown remarkable potential for automatically and non-invasively collecting phenotypic information. However, they also noted persistent technical barriers, such as visual occlusion, environmental complexity, and high implementation costs.

To address these challenges, specialized datasets and analytical models have been developed; for instance, an image dataset from a commercial poultry farm in China was assembled using a partially controlled capture methodology^3^. While this dataset contained highly accurate annotations, the limited environmental variability raised concerns about its ability to generalize to more diverse production settings.

Other efforts have targeted specific behavioral or welfare indicators. For example, Broiler-Net^4^ was introduced as a vision-based system for detecting inactivity and overcrowding in cage-free poultry houses. Yet, due to the absence of public datasets, this approach relied on internet-sourced videos, which inevitably reduced the system’s applicability to real production environments.

Similarly, deep convolutional neural networks have been employed to identify behaviors such as resting, feeding, and postural changes across different growth stages^5^. Although these models achieved promising accuracy, the controlled experimental environment limited generalization to commercial farms.

Other contributions have focused on resource use and counting tasks. Segmentation techniques have been applied to monitor how broilers use space and resources^6^, achieving robust results despite a small volume of annotated images.

High-density flock scenarios have been addressed through the creation of the Dense-Chicken dataset, which enables density-map-based counting networks to achieve low errors in congested settings^7^.

In parallel, the Chicks4FreeID dataset^8^ was developed for individual chick identification under neutral backgrounds and highly controlled conditions. While valuable for specific applications, its utility remains limited in complex commercial environments.

More recently, transformer-based architectures have entered the field. It has been demonstrated that these models can achieve real-time detection and tracking in long-term video studies^9^. However, the absence of truly intensive production conditions leaves open the question of how such systems would perform in the variable and high-density environments of commercial poultry farming.

Recent work in precision poultry farming highlights both the importance and the challenges of building reliable chicken object detection datasets under real commercial conditions. Complex environments such as caged laying hen houses introduce severe occlusion, uneven illumination, and crowded scenes, which significantly reduce detection accuracy. Similarly, multimodal approaches have emerged as a promising direction; for instance, a Transformer-based abnormal and dead hen detection framework combining image fusion with RT-DETR enhancements has demonstrated very high precision and mAP performance in low-light, crowded cage conditions^10^. Beyond detection, automated monitoring systems such as ChickTrack enable detection, counting, and tracking of individual birds for welfare assessment in realistic farm environments^11^. Despite these advances, the field still suffers from a lack of large-scale standardized open-access datasets and consistent benchmarks, as emphasized by a recent survey of 20 publicly available poultry vision datasets^12^. Together, these efforts underline the growing need for high-quality annotated datasets to support robust chicken detection models in precision livestock farming.

Taken together, these studies reveal a consistent narrative: while progress has been made, the field still lacks large-scale, openly available datasets that capture the variability, density, and environmental conditions of real-world poultry production. Without such resources, it remains difficult to compare methodologies, benchmark performance, or develop robust, scalable solutions for precision poultry farming. A comparative summary of the main publicly available datasets and their limitations relative to the present work is provided in Table 1.

**Table 1:** Comparative summary of relevant broiler chicken detection datasets.

Dataset	Annotation Type	Capture Environment	Env. Variability	High Density	Manual Annotation	Main Focus
^3^	Bounding boxes	Commercial (China), partially controlled	Medium	No	Yes	Accuracy in stable settings
ChickTrack^11^	Bounding boxes (Tracking)	Realistic farm videos	High	Yes	Yes	Welfare & activity tracking
^6^	Segmentation	Resource monitoring	Medium	No	Yes	Space & resource usage
^5^	Bounding boxes / Classification	Controlled experimental	Low	No	Yes	Behavior recognition
Dense-Chicken^7^	Density maps	High-density flock	Low	Yes	Yes	Congested counting
Chicks4FreeID^8^	Semantic segmentation	Controlled, neutral background	Low	No	Yes	Individual identification
Broiler-Net^6^	Bounding boxes	Internet-sourced videos	High	Yes	Yes	Inactivity & overcrowding
^9^	Bounding boxes (Tracking)	20-day video study	Medium	No	Yes	Real-time tracking
Present Work (2025)	Bounding boxes	Real commercial broiler farm	High	Yes	Yes	Scalable edge AI monitoring
^10^	Multimodal (Visible/Thermal)	Caged hen houses	High	Yes	Yes	Abnormal/Dead hen detection

It is against this backdrop that we introduce PIO, a brand-new large-scale dataset collected in both real commercial and experimental poultry houses^13^. Comprising 1,487 manually annotated images and more than 327,289 broiler instances, PIO encompasses high stocking densities (up to 500 birds per image), significant environmental variability, and multiple growth stages. By bridging the gap between controlled research environments and the complex realities of poultry production, PIO offers a robust foundation for object detection, counting, behavioral analysis, anomaly detection, and welfare assessment in automated monitoring systems.

## Methods

This study provides a detailed description of the design, collection, selection, and annotation of images comprising the PIO dataset^13^, developed for training object detection models in real poultry farm environments. The primary objective was to capture images representative of diverse operational conditions, including variability in lighting, stocking density, and growth stages, thereby ensuring a robust and versatile dataset.

The images were obtained from video recordings collected in two distinct physical facilities: a prototype poultry house and a commercial poultry house. Both locations reflect typical production environments, with the prototype facility representing a semi-controlled setting and the commercial facility reflecting a real high-density poultry production environment. Data collection was entirely passive, involving no physical contact or alteration of animal behaviour, in strict adherence to ethical principles of non-intervention.

### Bioethics approval

The research protocol was reviewed by the Institutional Research Bioethics Committee of the Universidad Tecnológica de Panamá and was granted exemption from bioethics evaluation under the identification number P-CIBio-056-2025.

## Data Collection

**Fig. 2:**
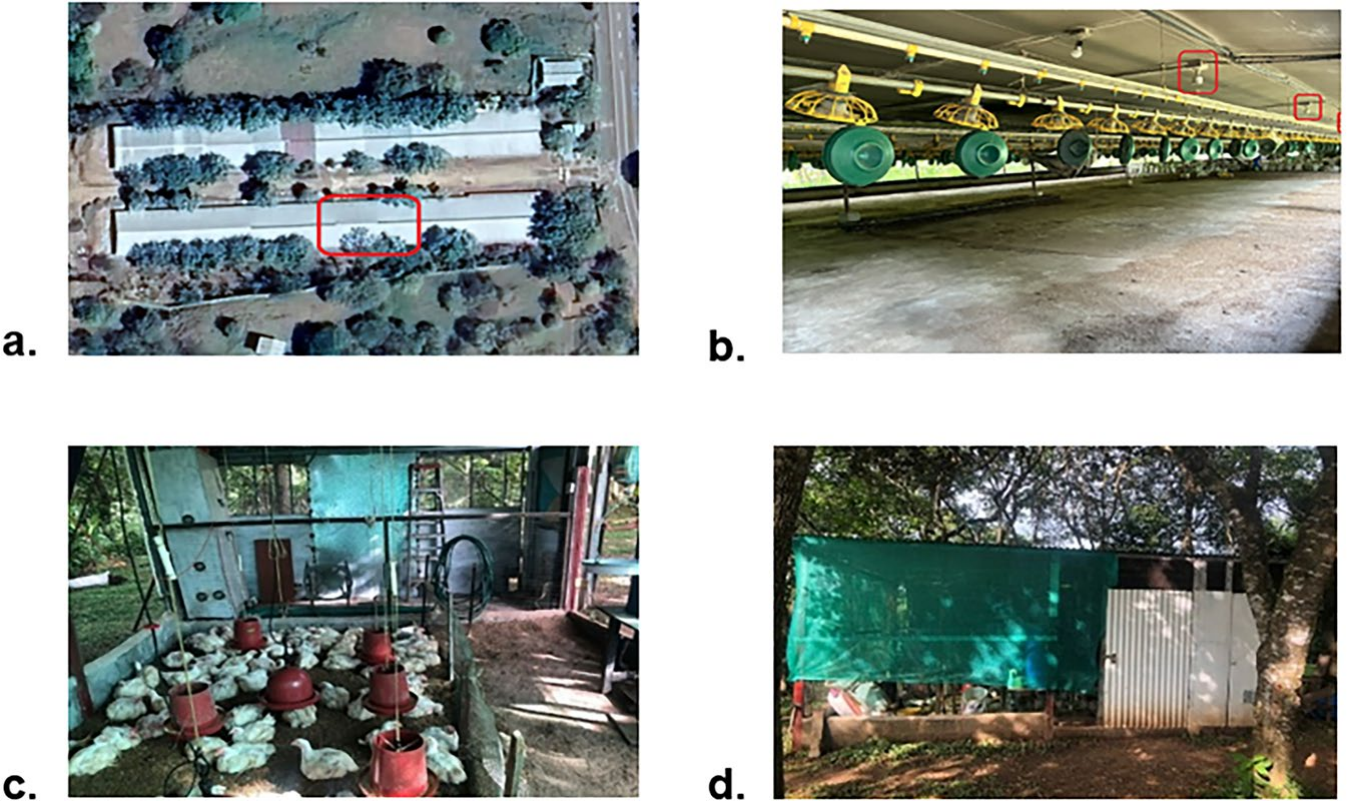
Representative views of the poultry houses used in the study: (**a**) aerial view of the commercial poultry house with the monitoring area highlighted in red; (**b**) interior of the commercial poultry house showing the feeding lines and installed cameras; (**c**) interior of the prototype poultry house with broiler chickens and manual drinkers/feeders; (**d**) exterior view of the prototype poultry house located in a forested area with natural ventilation.

The commercial poultry house corresponds to the facilities of El Buen Pastor farm, located in the province of Veraguas, Panamá, and represents a real production environment. In contrast, the prototype poultry house was built for experimental purposes in Quebrada Honda, Santiago, replicating similar conditions on a reduced scale. Both facilities were continuously monitored for 24 hours over a six-week period, covering the entire broiler growth cycle, as shown in Fig. 2.

The structural conditions of both poultry houses are summarized in Table 2. Key differentiating aspects include the number of cameras, stocking density, covered area, and type of lighting used. Ambient temperatures ranged from 22 °C to 32 °C throughout the capture period.

**Table 2:** Comparison of structural and monitoring conditions between the prototype and commercial poultry houses used in this study.

Parameter	Prototype poultry house	Commercial poultry house
Number of cameras	4	36
Approximate number of broilers	100	700 per frame
Area dimensions	6 m × 4 m (24 m²)	12 m × 8 m
Recording schedule	24 hours, continuous	24 hours, continuous
Type of lighting	Mixed (natural, artificial LED)	Predominantly natural, nighttime light, LED
Ambient temperature	22 °C – 32 °C	22 °C – 32 °C
Estimated stocking density in final stages	6–10 birds/m²	11–14 birds/m²

### Recording equipment and configuration

The capture devices were the Hikvision DS-2CD1027G0-L IP cameras that were used, featuring a native resolution of 1920×1080 pixels, F1.0 aperture, 24/7 colour vision, and H.265+ compression technology. Each unit is IP67-certified for harsh environments. The cameras were installed in an overhead (zenithal) position, at an average height of 2 meters above ground level, recording at 30 fps. This setup enabled full coverage of the space and precise capture of group behavior in the birds.

### Capture frequency and timing

**Fig. 3:**
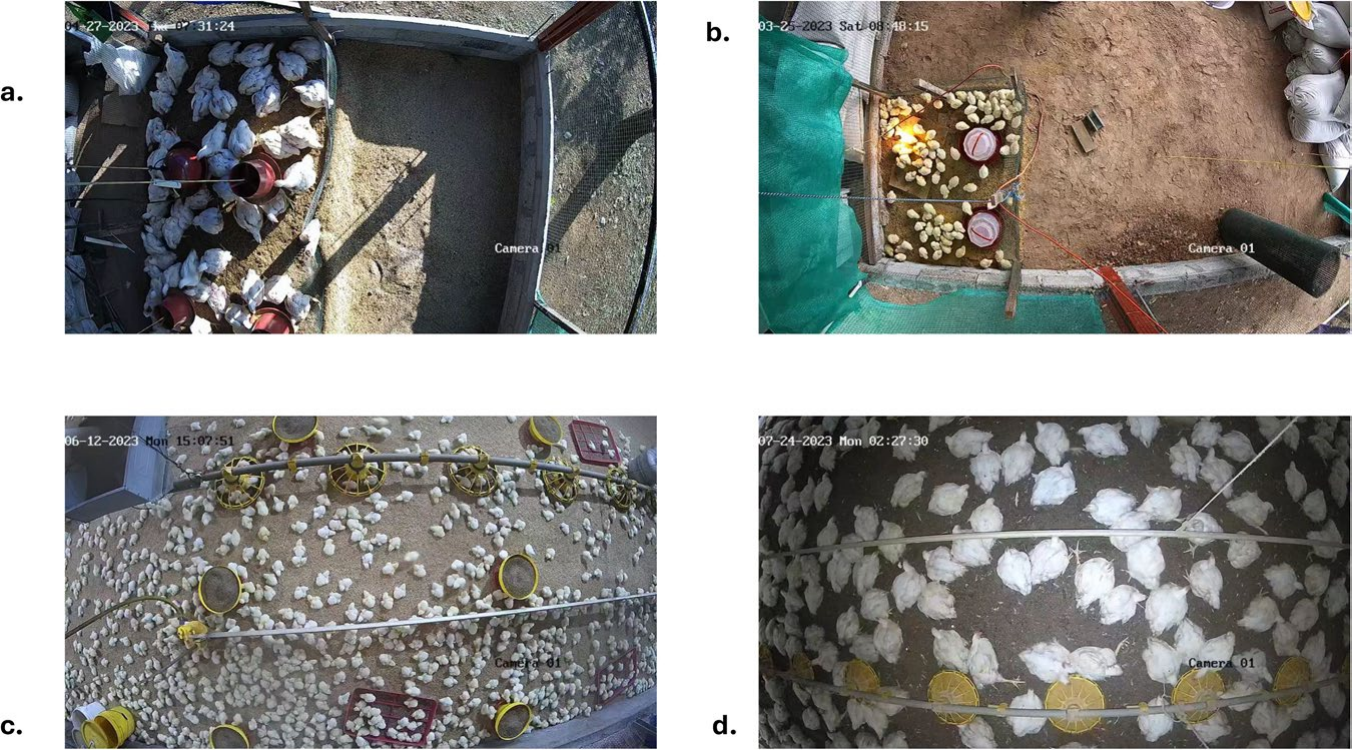
Representative views of broiler growth stages captured by overhead cameras: (**a**) intermediate stage in the prototype poultry house, showing clustering around drinkers; (**b**) initial stage in the prototype poultry house, with high mobility and dispersed distribution; (**c**) initial stage in the commercial poultry house, showing high density and movement; (**d**) final stage, with broilers clustered and exhibiting low mobility, which facilitates detection.

In both the prototype and commercial poultry houses, video capture was conducted continuously over 24 hours a day for a six-week period during 2023, covering the entire broiler production cycle from arrival to the final growth stage. This monitoring recorded a variety of behaviours associated with animal welfare, including feeding, resting, locomotion, and group interactions. Table 3 summarizes the stages of broiler growth, and Fig. 3 presents actual video frames obtained during these stages.

**Table 3:** Growth stages of broiler chickens.

Stage	Age (weeks)	Average weight	Relevant observations for detection
Initial	1	150 g	Increasing body size; crossings and rapid movements still occur.
Intermediate	2–3	0.4–1 kg	Increasing body size; crossings and rapid movements still occur.
Final	4–6	1.5–2.54 kg	Lower activity and larger body area.

#### Data selection

Image extraction from the original videos was performed using a Python script, where 250 frames per video were extracted with OpenCV and multiprocessing for parallel processing. The frames were exported in.jpg format and renamed following a structured convention.

Subsequently, blurred or duplicate content was removed, prioritizing visual diversity by considering different bird ages and sizes, variations in density and positioning, and the presence of contextual elements such as drinkers and shade nets.

## Data Annotation

The image annotation process for the poultry dataset was carried out during 2025. During this period, representative images from both the commercial and prototype poultry houses were carefully selected, considering different scenarios of stocking density, lighting, spatial arrangement, and various stages of broiler growth.

The images were manually annotated using the LabelImg^14^ tool, which allows the definition of bounding boxes around the objects of interest. For each image, an associated text file was generated containing one line per identified object, following the structure:


<class_id> <x_center> <y_center> <width> <height>


**Fig. 4:**
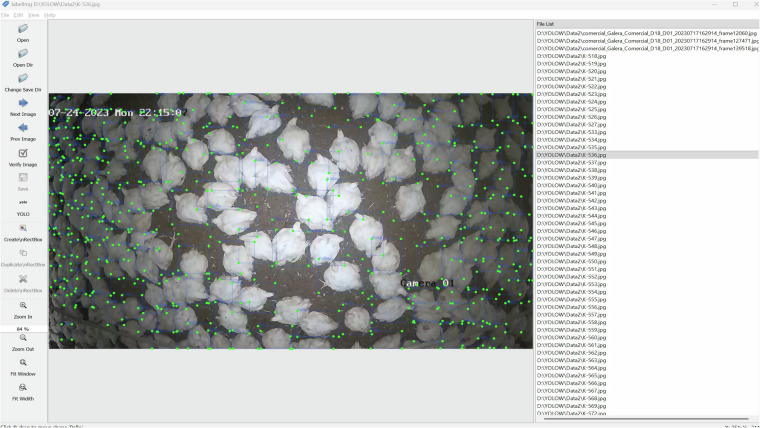
Manual annotation process using the LabelImg tool.

All values are normalized between 0 and 1 with respect to the image size. In this study, a single class (0) was used, corresponding to the broiler chicken, as shown in Fig. 4.

### Dataset Applications and Usability

The usability of this dataset is centered on its application for real-time monitoring in commercial poultry environments. Unlike datasets captured in controlled laboratory settings, these images reflect the lighting variations and dust conditions typical of broiler houses in tropical regions. This makes the dataset highly suitable for:

- Behavioral Analysis: Tracking movement patterns to detect early signs of illness or thermal stress.
- Population Density Monitoring: Developing automated systems to evaluate bird distribution and floor space efficacy.
- Edge and Standard AI Development: This dataset is a robust benchmark for training lightweight models optimized for Edge AI platforms and for developing complex architectures in standard high-performance computing environments for large-scale poultry research

## Data Records

**Fig. 5:**
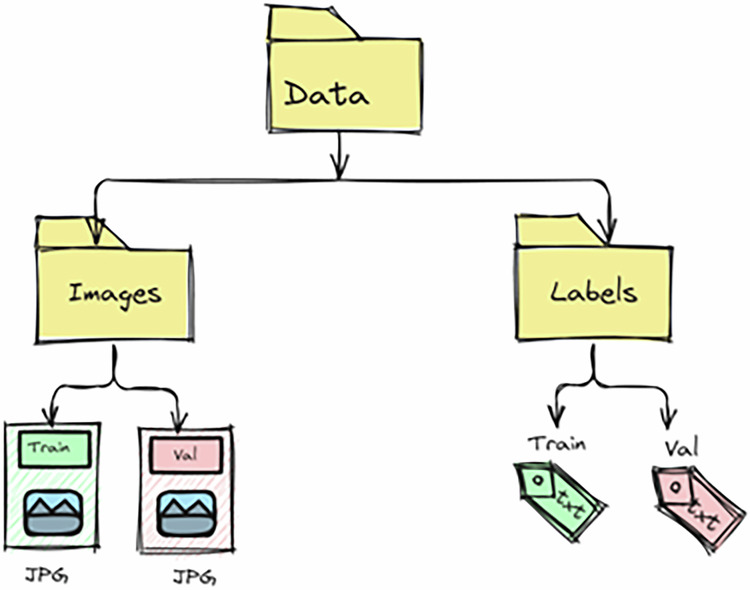
Folder structure of the annotated dataset.

The final dataset consists of 1,487 annotated images containing a total of 327,289 instances. The database was divided into two subsets: 70% of the images were allocated for training and 30% for validation, ensuring that both environments were represented in each partition. All files follow a structured naming convention to guarantee direct correspondence. The structure of the annotated dataset is illustrated in code 1 and Fig. 5.

**Code 1**. Folder structure of the PIO annotated dataset.


/images/train/val/labels/train/val


Each .txt file contains multiple lines representing the instances detected in a single image. The complete dataset includes a total of 327,289labelled instances. The data are ready to be used directly in YOLO11-based^15^, training workflows, including a *dataset.yaml* file that defines the image and label paths as well as the list of available classes. The descriptive metadata of the PIO dataset^13^ are summarized in Table 4 which provides details on dataset composition, source, collection and annotation periods, data formats, annotation structure, and ethical considerations to support reproducibility and responsible use.

**Table 4:** Summarizes what the dataset contains and under which conditions.

Metadata Element	Description
**Dataset name**	PIO: Broiler chicken detection dataset from commercial and prototype poultry houses
**Dataset description**	Contains 1,487 images with a total of 327,289 manually annotated instances (broiler chickens).
**Data source**	Videos recorded in two poultry houses: one commercial facility and one prototype experimental monitoring house.
**Collection period**	2023
**Data format**	.jpg and .txt files. Resolution: 1280×720 pixels.
**Annotation period**	2025
**Annotation structure**	.txt files with normalized bounding boxes per image: <class> <x_center> <y_center> <width> <height>
**Ethical and contextual considerations**	The dataset is based on real images of broiler chickens in both commercial and experimental production environments. Responsible use of the data in animal welfare contexts is strongly encouraged.
**Collaboration and usage**	Researchers are encouraged to use the dataset for developing detection, monitoring, and behaviour analysis solutions, fostering ethical and academic collaboration.

**Fig. 6:**
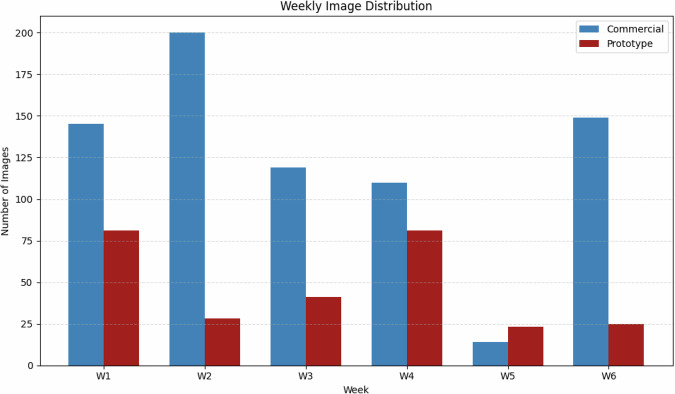
Weekly Image Distribution.

Figure 6 presents a bar chart illustrating the weekly distribution of annotated images in the PIO dataset^13^, categorized by poultry house type: commercial and prototype. Each week (W1 to W6) is represented by a pair of adjacent bars corresponding to each environment. A higher proportion of images were collected from the commercial poultry house, particularly during weeks W1, W2, and W6. This selection was deliberate, as these weeks exhibited greater variability in visual conditions—such as high chick density, overlapping individuals, uneven lighting, and structural elements causing occlusions. By prioritizing data collection under such challenging scenarios, the aim was to train detection models that are more robust to disruptive conditions, thus better reflecting the real-world complexities of intensive poultry farming systems. In contrast, the prototype poultry house offered a more controlled and visually homogeneous environment, which, while useful for comparative testing, posed fewer challenges for automatic detection.

**Fig. 7:**
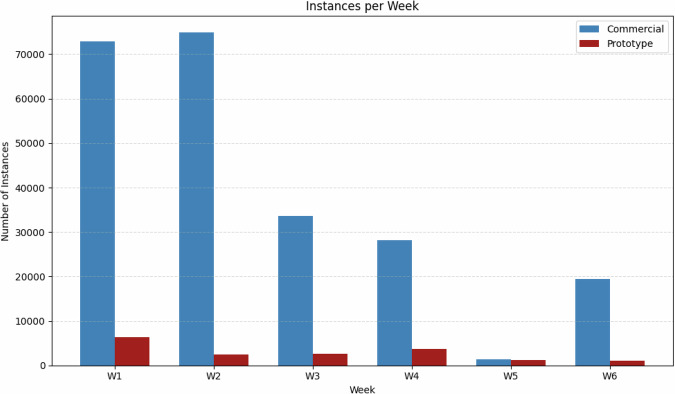
Instances per Week.

Figure 7 displays the weekly distribution of the total number of annotated chick instances in the PIO dataset^13^, distinguishing between images captured in the commercial poultry house and those from the prototype facility. Weeks are labeled W1 through W6, corresponding to different stages in the broiler growth cycle.

A notable concentration of instances is observed in the commercial house during weeks W1, W2, and W6. This pattern reflects a deliberate data collection strategy focused on capturing images under more challenging visual conditions, including high chick density, overlapping individuals, occlusions, and varying lighting conditions. Such scenarios are important for training robust and generalizable detection models capable of handling the complex visual environments typical of commercial poultry production systems.

## Technical Validation

To support the technical validity of the dataset, inference tests were conducted using the YOLOv10^16,17^ model on an unseen subset of validation images. The images used for this evaluation correspond to records obtained in 2025 from a representative flock in the commercial poultry house, captured under real production conditions.

Technical validation of the dataset was carried out through an experiment with a convolutional neural network–based object detector. YOLOv10 was selected due to its well-recognized effectiveness in fast and accurate detection scenarios. The hardware and software specifications of the experimental environment are detailed in Table 5.

**Table 5:** Specifications of the hardware environment used for training.

Component	Specification
**Processor (CPU)**	Intel® Core™ i9-14900HX (2.20 GHz)
**RAM**	32 GB
**GPU**	NVIDIA RTX 4060 (8 GB VRAM)
**Framework**	PyTorch 2.1.0
**Model**	YOLOv10
**CUDA version**	12.6

### Evaluation of performance metrics

The performance evaluation of the models on the PIO dataset^13^ was carried out using standard object detection metrics: precision, recall, and F1-score. These metrics have been widely validated in the specialized literature, where the implications and limitations of different evaluation approaches in computer vision are thoroughly discussed^18^. The evaluation metrics used in this study, including precision, recall, F1-score, and mean Average Precision (mAP) at different IoU thresholds, are summarized in Table 6.

**Table 6:** Mathematical definitions and technical description of the evaluation metrics used in the dataset validation.

Evaluation Metric	Symbol	Equation	Technical Description
1. Precision	*P*	$P=\frac{{TP}}{{TP}+{FP}}$	Proportion of correctly predicted positives over the total number of positive predictions.
2. Recall	*R*	$R=\frac{{TP}}{{TP}+{FN}}$	Proportion of true positives identified with respect to the total number of objects present.
3. F1-score	*F₁*	${F}_{1}=2\cdot \frac{P\cdot R}{P+R}$	Harmonic mean of precision and recall, summarizing the balance between both metrics.
4. mAP@50	mAP@50	${{mAP}}_{50}=\frac{1}{N}\mathop{\sum }\limits_{i=1}^{N}{{AP}}_{i}\circ \left({IoU}\ge 0.50\right)$	Mean Average Precision across all classes, considering a correct prediction if IoU ≥ 0.50.
5. mAP@50-95	mAP@50-95	${{mAP}}_{50:95}=\frac{1}{10}\mathop{\sum }\limits_{k=1}^{10}{{\rm{mAP}}}_{{{\rm{IoU}}}_{k}}\circ ,{{\rm{IoU}}}_{k}\in \{\mathrm{0.50,0.55},\ldots ,0.95\}$	Mean Average Precision computed at 10 IoU thresholds (0.50 to 0.95). Measures overall model performance across varying detection strictness levels.

### Hyperparameter configuration

To validate the utility of the proposed dataset for object detection tasks, three variants of the YOLOv10 model were trained: YOLOv10n, YOLOv10s, and YOLOv10m. The training process was conducted in a Linux-based environment using Ultralytics version 8.3.152 and an NVIDIA GeForce RTX 4060 GPU. All models were trained for 100 epochs, as summarized in Table 7.

**Table 7:** Hyperparameter configuration used for training with YOLOv10n, YOLOv10s, and YOLOv10m.

Parameter	YOLOv10n	YOLOv10s	YOLOv10m
Image size	640 × 640	640 × 640	960 × 960
Learning rate	0.002	0.002	0.02
Optimizer	AdamW	AdamW	AdamW
Batch size	4	4	2
Momentum	0.9	0.9	0.9
Epochs	100	100	100
Instances	82,357	82,357	82,357

Table 8 compares the performance of two YOLOv10 model variants, specifically YOLOv10s and YOLOv10m, in terms of key training and evaluation metrics. YOLOv10m shows lower training losses (box_loss, cls_loss, and dfl_loss), indicating better model optimization. Moreover, YOLOv10m outperforms YOLOv10s in precision, recall, and accuracy-related metrics such as mAP@50 and mAP@50–95, suggesting superior object detection performance on the validation set.

**Table 8:** Performance comparison between YOLOv10s and YOLOv10m models.

Metric	YOLOv10n	YOLOv10s	YOLOv10m
Box loss (*box_loss*)	2.1	1.9	1.6
Class loss (*cls_loss*)	0.9	0.8	0.6
DFL loss (*dfl_loss*)	1.95	1.9	1.75
Precision	**0.938**	**0.954**	**0.961**
Recall	0.84	0.86	0.88
mAP@50	0.90	0.92	0.97
mAP@50–95	0.671	0.70	0.76

### Visualization

To support the technical reliability of the dataset, inference tests were performed using the YOLOv10s and YOLOv10m models on an unseen subset of images. These images were obtained in 2025 from the commercial poultry house El Buen Pastor in Veraguas, Panama. The resulting predictions enabled a visual assessment of the correspondence between the manual annotations and the detections generated by the trained models.

**Fig. 8:**
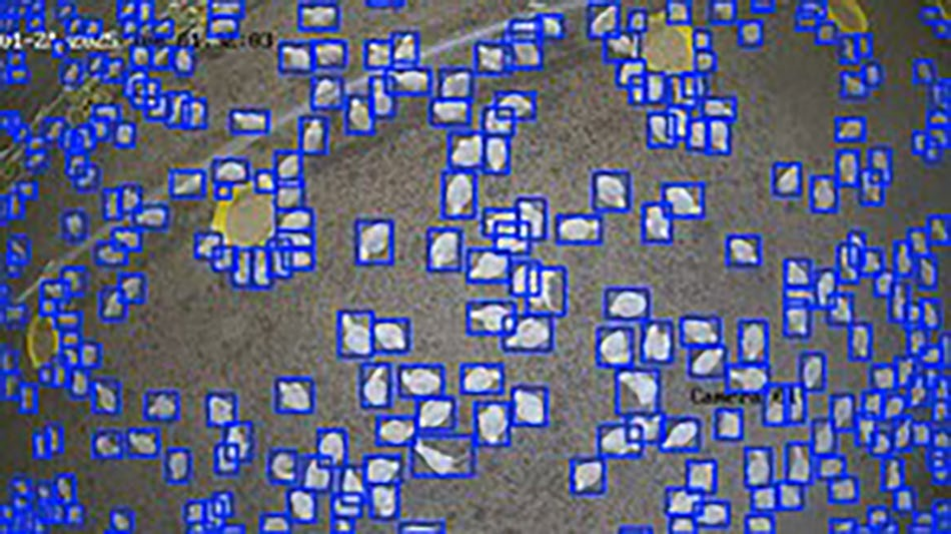
Visual results of the YOLOv10n model on an image from the El Buen Pastor commercial poultry house.

**Fig. 9:**
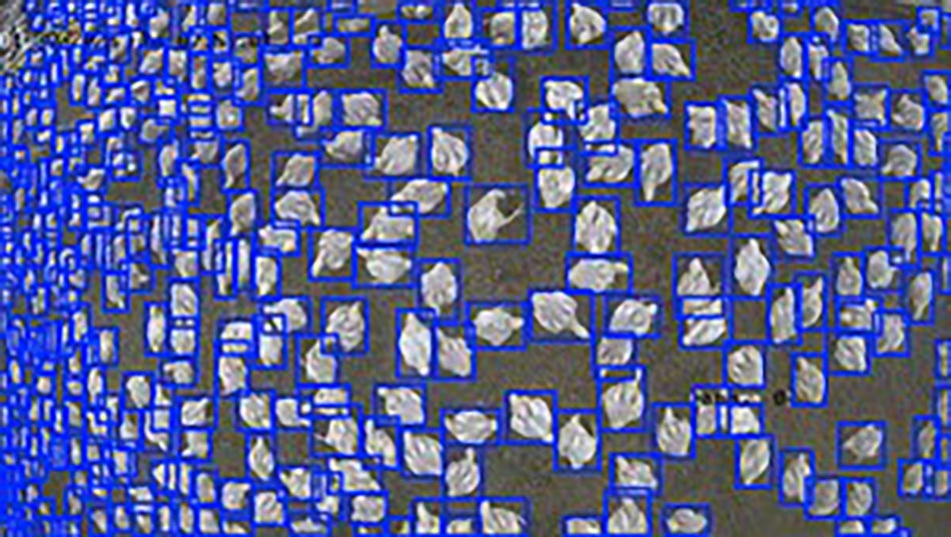
Visual results of the YOLOv10s model on an image from the El Buen Pastor commercial poultry house.

**Fig. 10:**
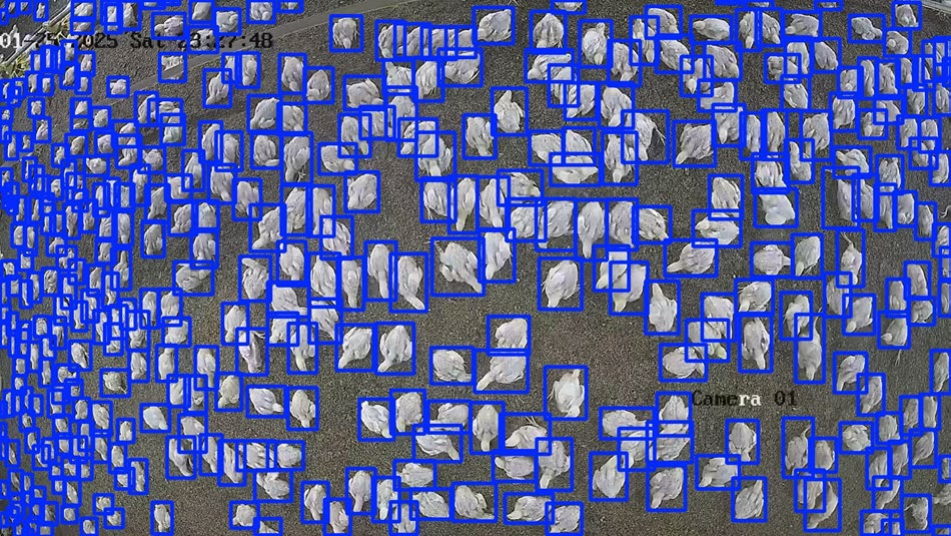
Visual results of the YOLOv10m model on an image from the *El Buen Pastor* commercial poultry house.

The visualizations show correct overlap between the predicted bounding boxes and the objects of interest, even in scenes with high density, morphological variability, and heterogeneous lighting conditions. These qualitative observations demonstrate that the dataset contains consistent and suitable annotations for training object detection models, thereby supporting its technical quality and applicability in computer vision contexts for poultry farming environments. Figures 8, 9 and 10 illustrate examples of model predictions under the aforementioned real conditions.

## Data Availability

The Dataset is available at ZENODO^13^ with the URL 10.5281/zenodo.16686320. The dataset includes: **• Images**: 1,487 manually annotated images from prototype and commercial poultry houses in Panama. **• Annotations**: YOLO-format text files containing normalized bounding boxes for broiler chickens. **• Metadata**: Documentation detailing acquisition conditions (lighting, stocking density, growth stage, facility type). • Folder structure: ◦ /images/train – training set images ◦ /images/val – validation set images ◦ /labels/train – corresponding annotations for training images ◦ /labels/val – corresponding annotations for validation images Additionally, a folder with original videos from the poultry houses is provided, allowing the research community to continue labeling, expand the dataset, or explore new computer vision tasks.

## Code availability

The preprocessing scripts, annotation analysis tools, and training pipelines are hosted in the companion GitHub repository: https://github.com/YESUBZERO/MONZAD-OPI5.
